# Fostering trustworthy information: countering disinformation when there are no bare facts

**DOI:** 10.1098/rsos.250654

**Published:** 2025-06-18

**Authors:** Marcel Boumans, Joras Ferwerda, Maya J. Goldenberg, Sabina Leonelli, Federica Russo, Vincent Traag, Arjan Wardekker

**Affiliations:** ^1^ Utrecht University, Utrecht, The Netherlands; ^2^ University of Guelph, Guelph, Ontario, Canada; ^3^ Technische Universität München, Munich, Germany; ^4^ Universiteit Leiden CWTS, Leiden, The Netherlands; ^5^ University of Bergen, Bergen, Norway

**Keywords:** disinformation, fact-checking, trust in science, uncertainty, validity

## Abstract

At a time when the dissemination of online information is synonymous with an abundance of disinformation and misinformation, it is important to extend our reflection beyond debunking and fact-checking. In this article, we consider the cases of (dis-, mis- and mal-)information regarding scientific results. We argue that countering misinformation requires a better understanding of the root cause of the problem. We believe the root cause is trust rather than truth. We argue that trust should be approached from a distinct social epistemological perspective that recognizes differences between data and facts and that treats trust as part of the scientific process and as part of the way publics interpret and use scientific information.

## 1. Truth or trust

As the dissemination of online information has become synonymous with an abundance of disinformation and misinformation, we feel the urgency that it is time to move beyond modes of combating it such as debunking and fact-checking. We believe that combating disinformation will be more effective if it is distinguished from fact-checking, in the sense that trustworthy information is distinguished from the concept of truth: information is not trustworthy because it is true, but rather because it follows from trustworthy procedures.^1^ In this article, we focus on disinformation and misinformation related to scientific results.

Originally, the meaning of the term ‘information’ is derived from the verb to inform which means ‘to train, instruct, educate in some specific subject’ [1]. Its current meaning is ‘the imparting of knowledge in general’ [2]. The meaning of the term ‘information’ is in these definitions not associated with a specific reference to the truth.^2^ In line with these definitions, we consider information to be highly contextual and dependent on both given data (i.e. measurements, observations and statements) and methods through which such data are interpreted and contextualized. In other words, information is the way in which data are imbued with meaning, which in turn places the emphasis on the skills used to identify and understand information as much as it does on the elements that contribute to information in the first place (such as data and observations).

The problem with fact-checking is that it is based on the assumption that there is knowledge that is not contextualized and for which we can find a unique correspondence with facts in the world that makes it true [3]. It takes knowledge as ‘bare facts’ and that its veracity can be established in a straightforward way. However, what is considered fact emerges from a trustworthy process of evaluating trustworthy data. Such trust is gained by opening the black box of how information is distilled from data and noting how important contextualization and interpretation are in that process.

More attention needs to be paid to the context in which information is evaluated and understood as trustworthy, particularly in terms of whether, how and when information can be trusted. Moreover, to understand information in its relationship to trust, we must develop a concept of trust that is not just based on correspondence to truth or on accuracy (i.e. closeness to the truth—which can be difficult to ascertain) but that is grounded in the conditions under which information is generated and promulgated.

We propose to consider disinformation as originating from sources that lack procedures for trustworthy production, interpretation and dissemination of information in science communication context. The problem of disinformation is then characterized by a misdirection of trust and misguidance of the grounds on which trust is attributed. We believe that we need to combat disinformation based on the restoration of trust rather than by adjudicating ‘truth’. In short, combating disinformation is more effective when it is discussed not in terms of truth but in terms of trust, where trust is discussed in terms of trustworthiness and reliability. This article explores these relationships to trust, where they are disrupted and where the possibilities for recovery lie.

In this article, we consider the cases of disinformation regarding scientific results [4]. We argue that decisions on countermeasures require a better understanding of the root cause of the problem, which we identify as lack of trust in science. Even though most people generally trust science [5], certain ideological orientations are associated with a lower trust. We argue that trust should be approached from a distinct social epistemological perspective that recognizes differences between data and facts, and that treats trust as part of the scientific process and as part of the way publics interpret and use scientific information [6]. The social epistemological approach of this article implies a ‘situational approach’ which recognizes that the establishment and acceptance of facts are based on trust and that facts reflect inherently social relationships and are not objective objects. Such situational understanding of misinformation involves a less victorious and heroic image of science, grounded on acknowledging that scientists do not always get it right and that attempts to constantly challenge and improve what is assumed to be true are at the core of scientific methods [7].

Although trust in science has been studied intensively by philosophers of science, historians of science and social scientists, usually under the umbrella of the relationship between science and society, their research does not seem to play a major role in the discussion of how to counter disinformation.^3^ One possible reason could be that fact-checking strategies are rooted in a naive positivist image of science, which assumes that a clear-cut distinction can be made between fact and fake. We believe that a better understanding of how facts are made will help restore the relationship of trust between science and society.

## 2. The need for a different approach to combating disinformation

Before we open the black box of how facts are obtained from data, we first argue why we think such an exercise is necessary. The dissemination of information has existed at least as long as relevant media have existed. The rate of increase, as well as qualitative changes in its spreading, is often linked to technological developments. For example, the invention of printing not only led to exponential growth in the distribution of books but also of pamphlets and tracts. These prints were often intended to discuss or question authorities such as the church or monarchs, or to spread information hidden by state, church or other powerful authorities. So even if they might contain disinformation, they were crucial to the establishment of liberal democracies. The spread of these materials was crucially supported and motivated by the enactment of large public education programmes, increasing literacy across European societies and therefore opportunities for broader publics to engage with such materials. With the introduction of *digital* technologies, and especially with the introduction of social media as vehicles of information exchange, we have not only witnessed a tremendous increase in the speed of information circulation, but we have also seen the emergence of new forms of interactions that lead to a need to understand the world in informational terms and devise education and training programmes to match that need [9]. What is unprecedented, however, is the current scale of information manipulation, as it can be produced by generative artificial intelligence (AI).

Moreover, what is called foreign information manipulation and interference (FIMI) has become a real threat, not only to the functioning of young liberal-democratic societies. FIMI includes the dissemination of disinformation but places emphasis on patterns of behaviour that threaten or have the potential to negatively impact democratic processes, security and citizens [10]. Such programmes build on the gap in skills and understanding between technology developers and prospective users by providing ready-made and one-dimensional interpretations that may distort the significance of the information being disseminated. To give an idea of the scale of this, in 2022, the Russian Federation contributed 1.5 billion USD from the federal budget for mass media. According to Debunk.org analysts Michałowska-Kubś & Kubś [11], this amount has probably been exceeded and they estimate that state-owned or state-sponsored mass media spent around 1.9 billion USD in 2022.

Whether it concerns foreign or domestic information manipulation and interference, these technology-driven developments disrupt the functioning of liberal democracies by challenging shared understandings of truths and facts. Both epistemic realists and social constructivists agree that the functioning of liberal democracies depends on reliable, trustworthy knowledge—regardless of the ontological status of ‘truth’. Shared facts are the common ground upon which political discourse and accountability can occur. Political accountability is impossible if facts can be evaded by relabelling them as ‘fake news’ or substituting them with ‘alternative facts’.

The gravity of the situation is well recognized. Numerous interventions have been developed and organizations established: for debunking disinformation or fact-checking, we have organizations such as the international DebunkEU.org, the EU East StratCom Task Force, Duke’s Reporters LAB and the Dutch Nieuwscheckers.nl. For boosts, educational interventions and nudges, we can mention for example the US-based Center for Media Literacy’s online Reading Room and Media & Values Archive.^4^ But despite this recognition and the many good interventions that have been developed, we believe that they are still insufficient and that a social approach to knowledge (i.e. social epistemology) is needed that distinguishes disinformation from facts and focuses on the ways in which information is interpreted and trusted. In other words, we need to shift our focus from truth to trust. There are four reasons to argue for such an approach.

First, the fight against disinformation cannot be won by simply pouring more money into debunking. In 2018, the East StratCom Task Force was granted €1.1 million from the European Parliament’s Preparatory Action for its work to address pro-Kremlin disinformation; in 2019, this budget increased to €3 million and in 2020 to €4 million; and for 2021, the overall budget devoted for the European External Action Service (EEAS) Strategic Communications and Information Analysis Division to address disinformation and manipulative interference and strategic communications capabilities equals €11.1 million [13]. This is a factor of 1000 less compared with the Russian state budget on mass media. One could aim for a strategy of an arms race, but as the term suggests, this is most likely to lead to more arms, not their reduction. Moreover, it remains much cheaper to produce disinformation than to debunk it. While greater investments are certainly necessary, we must come up with a different approach that compensates for the gap in this arms race.

Second, many strategies are post hoc. Investments tend to focus on identifying and debunking disinformation, which by definition happens afterwards and not in advance. There are also potential ‘backfire effects’ in which corrections increased belief in the targeted misperception among groups that were predisposed to believe the claim [14], although the effect is also debated [15]. Indeed, not all disinformation might require active debunking, and responding might just provide ‘oxygen’ to disinformation [16]. Moreover, the harm caused by disinformation cannot always be undone even if corrected information is widely disseminated and eventually accepted: neutralizing fake does not neutralize the harm. In fact, in the digital sphere, harm can persist even if the original source of harm is removed or made digitally inaccessible, as the debate around the ‘right to be forgotten’ has shown [17].

Third, the reach of fact-checking strategies is limited. Debunking, while important and useful, appears to be very limited in its efficacy. Not only because it is impossible to debunk or fact-check at the same pace as disinformation spreads, but also because fake news cannot be reduced to bare facts but are instead couched in arguments and supported by values. For example, misinformation about the harms of public health measures during COVID was compelling to information seekers who worried about government overreach and/or infringement on individual liberties. In other words, disagreements over the trade-offs associated with lockdown measures and/or mandatory vaccination were a big part of the so-called infodemic. Countering disinformation is not just a matter of providing different or better information but also of augmenting people’s argumentation and literacy skills to facilitate critical debate over specific beliefs, especially as these skills can bring values to the fore [18,19]. Indeed, the idea of addressing an ‘information-deficit’ is not necessarily effective [20]. The advent of generative AI expands the challenge of critically assessing not only textual information but also audiovisual information.

Fourth, delineating information from disinformation is not straightforward. This is mainly due to the lack of a clear conceptualization of disinformation. Current actions to counter disinformation are based on the distinction between information and disinformation, where disinformation is defined by the European Union as ‘verifiably false or misleading information that is created, presented and disseminated for economic gain or to intentionally deceive the public, and may cause public harm.’^5^ The problem with this definition is twofold. First, it limits actions to checking facts and then correcting the verified falseness of information, with the disadvantages mentioned above. Second, the distinction between information and disinformation is based on a difference in *intent*, i.e. whether or not to deceive or mislead for economic or political gain, simply understood as ‘bad intentions’. The problem with this distinction between disinformation and information is that, as we will show below, information can be (unintentionally) wrong, and so the core of the distinction is based solely on whether the intentions were good or bad. But intentions are often unclear or obscured, so according to the EU definition, you can only counteract their harm if the intentions are made explicit or clear; then one can block its source. But if the intentions are obscured one cannot reduce the harm until they have become visible and perceivable. Obscured bad intentions can only be detected through backwards inference, that is, when the harm has been done, by reasoning backwards as to why it was done. Moreover, harmful information can also be spread through good intentions, such as commitment to public health or the well-being of one’s neighbourhood—as clearly shown by beliefs such as vaccines causing infant death and 5G networks spreading cancer, which are often fuelled by concern for the negative effects that such innovations may have on one’s community.

Finally, the definition implies a problematic verifiability of falseness, which is the underlying idea of fact-checking. Although there is merit in trying to establish the accuracy of any claim, this is not straightforward. Although some misinformation around facts might be easy to resolve and mark as ‘misinformation’, in other cases, it might be nearly impossible to unequivocally state that something is ‘wrong’ because of uncertainties. Making claims is easy, but establishing the truth is very difficult. Sometimes information might not even be categorically wrong but just inapplicable to the case at hand (sometimes labelled malinformation), and such strategies may even be intentionally used to sow doubt [22]. So, establishing the veracity of any single piece of information does not necessarily address the problem that comes with that information.

To clarify the difficulty of deciding with certainty whether some piece of information is true, it is useful to distinguish between mistake and error. According to Hon [23], ‘error’ may be traced to the Latin root ‘errare’, which originally had two contrasting meanings: first, ‘to go this way and that, to walk at random’; and second, ‘to go off the track, to go astray’. So right at the origin of the term, there is a tension between aimless wandering and straying from some definite path. To make this tension more explicit, Hon makes a distinction between two ways of going wrong, which he calls, respectively, the way of *error* and the way of *mistake*. Mistake is avoidable ignorance. A mistake can be avoided since checking procedures are known and available. By contrast, he associates error with unavoidable ignorance, when one does not have the security of a well-studied, agreed standard procedure—when one gropes, so to speak, in the dark. We often do not know whether we have made an error, and this cannot always be avoided.

Because the interpretation of data, and therefore of what may constitute information, can be wrong, we need standard procedures to reduce the mistakes in it. In other words, to distinguish misinformation from information we need to know what standards to follow. These standards are not universal, each scientific field or context has its own standards, and they can shift over time. Moreover, there are differences in views within and outside the sciences about what these standards are. Therefore, one way to understand information in its relationship to trust is to better understand the standards used to verify the reliability of information; that is, to avoid mistakes.

## 3. The difference between data and facts

An epistemological account that helps clarify the production of facts in natural science, or more generally in any science, is Bogen & Woodward’s ‘Saving the Phenomena’ [24]. In their view, facts about phenomena are not directly observable but ‘detected’ through the processing of data. Yet data, in the sense of observations and/or measurements generated to document a specific phenomenon, are *idiosyncratic* to the particular context in which they are produced.

Recognizing this distinction between data and facts implies two problems for which one must find a solution. The first can be called the problem of induction. The function of statistical inference is to go beyond the observed instances, thereby seeking to extend the description of certain characteristics of observed phenomena to more general facts about these phenomena. In other words, the purpose of statistical inference is not merely descriptive, that is, to provide a description or a summary of a dataset. It is also to postulate something more general. The induction problem is how to substantiate this generalization.

The second problem is how to get rid of the idiosyncrasy of the data, such that the data can play the role of evidence for claims about phenomena. To deal with this problem, Woodward proposes to characterize the distinction between data and phenomena in terms of the notions of error applicable to each [25]. In the case of data the notion of error will involve ‘perceptual or recording mistakes – misreading a dial or transposing digits when a number is entered into a laboratory notebook – or the outright manufacture of data, as in fraud’. In the case of factual claims about phenomena, the main concern is whether one is detecting a real effect rather than an artefact produced by peculiarities of one’s instruments or detection procedures or a bias in the statistical analysis. Empirical research is typically carried out in a ‘noisy’ environment. The problem of detecting a phenomenon is the problem of separating signal and noise in a reliable way.

To go beyond the observed instances to more general facts about the phenomena the data must be modelled, that is to say, the inference is model-based [26]. While Woodward discusses the separation of signal and noise in terms of experimental design and control, most sciences must work outside of controlled experimental settings. The lack of control and systematic intervention in most sciences is compensated by mapping the phenomenon and its environment into models that function as a representation of the phenomenon in question, which makes it possible to study it and generate knowledge about it. These models—usually statistical models—function as virtual laboratories, that is, as artificial worlds in which we can experiment [27].

Models, particularly when they come in the form of mathematical constructs, have been controversial since they became standard research practice in science in the mid-twentieth century. In our view, the main reason is that they are confused with theories. Theories are expected to tell the truth about the mechanisms and processes that exist in the world. Models, however, are better thought of as epistemic mediators, as Morgan & Morrison [28] have proposed in their book *Models as Mediators*. Mediating between data and theories, models are instruments of investigation, and each model is built to answer a specific question. And not all questions are about what the real processes and mechanisms are: often models are used to explore possibilities and alternatives, leaving questions around truth-value momentarily aside. The consequence of this distinction between theories and models is that models are tested in a different way. A model is tested on the quality of the answers it provides, that is, on how well it performs as an instrument, i.e. its ‘fitness-for-purpose’.

The recognition of facts as model-based processed data shifts the question of trust in scientific information to trust in models or other epistemic mediators, such as experiments and simulations. What are the standards for verifying the reliability of these mediators?

## 4. Validating scientific results

The validity of a model is understood as its usefulness with respect to a given purpose, that is, how well it is able to answer the question it is built for. To test them, models are asked questions to which the model-builder knows the answer. Examples of these validation procedures are Turing tests and calibration [29]. If the model reproduces these answers satisfactorily, we trust the model to answer questions (within the domain for which the model was developed) to which we do not know the answer.

In fact, the main epistemic requirement of models is that they are good simulators and that they produce good simulations. It is rather unfortunate that the term simulation has become the term to indicate or clarify the role of models in research, since this term has its origin in biology, and so its first meaning is ‘the action or practice of simulating, with intent to deceive; false pretence, deceitful profession’. A better term would have been ‘simulacrum’: ‘something having merely the form or appearance of a certain thing, without possessing its substance or proper qualities’ [30]. Nevertheless, both terms indicate that the model of a phenomenon is not the phenomenon itself but is the epistemic mediator for studying the phenomenon. That is why Morgan & Morrison call a model an ‘instrument of investigation’ [28].

Another way to ‘test’ models is to critically examine the way in which they were produced, including the choices made by the modellers, and the solidity, backing and alternatives for these. For example, in model-based assessments, analysts have to make assumptions. These inevitably involve judgements that are to some degree subjective and potentially affected by the analysts’ disciplinary, socio-political or practical values [31]. Assumption analysis can be used to critically assess these assumptions on potential value-ladenness and their impact on the results [32]. Other tools include, for instance, sensitivity analysis [33] and model quality checklists [34]. These types of tests aim to identify potential weak spots in the modelling process, limitations and strengths of the models (what they can and cannot tell the user with some degree of certainty) and potential avenues to improve them.

The artificiality of models is perhaps the reason to argue that experiments offer greater epistemic power than models as a means to investigate the world.^6^ This outcome rests on the distinction that while experiments are *versions of* the real world, models are artificial worlds built to *represent* the real world [36]. But the problem with experimentation is that they are captured within a laboratory environment that is still an artificial world in the sense that it is designed and created, a constrained version of reality. The conditions under which the experiment takes place must be fixed—the so-called ceteris paribus conditions—and known. Therefore, to verify that an experimental result cannot be just an artefact of the particular detection techniques we employ or of the local environment in which we operate, reproducibility is a crucial requirement.

Reproducibility is defined in terms of controlled conditions, which include procedures, operators, operating conditions, location and object of investigation, and time. If they are all the same except for time, and so reproduction is just ‘repetition’, this does not increase trust in the experimental results. One has to vary these conditions. But the question is which conditions should be varied and to what extent in order to increase trust but not lose the ‘integrity’ of the results, where integrity means that the factual content of both results is the same.^7^ Given this variation, an uncritical pursuit of reproducibility as an overarching epistemic value is misleading and potentially damaging the trust in results.^8^ Moreover, with publication bias on positive results, even incorrect results may become considered facts [40].

The problem with facts, whether as outcomes of models or as experimental results, is that the usual tools for testing the reliability of facts are actually trust-inducing strategies: we trust a model because it also reproduces known facts, and we trust an experimental set-up because it reproduces similar facts under different circumstances. What matters is the criteria used to assess the plausibility of research design and procedures, which are in turn used to evaluate the truth-value of the resulting claims [41,42]. Besides the well-known insight that science has no rock bottom to ground factual claims, an additional problem is that science can be imitated based on these criteria. As a consequence, fake that passes the same tests cannot be distinguished from fact. Fakes can be very good imitations of facts. Many examples of this situation can be found in bona fide scientific research, where outcomes that were long believed to be factual turned out to be false once the procedures used to generate and support them were scrutinized with particular intensity. The so-called Spidergate is a case in point, with results initially believed to be accurate about a particular spider species turning out to be unreliable and generating a domino effect in the entomological community as the web of trust built around those studies threatened the credibility of subsequent studies grounded on those initial results [43]. Similar issues are highlighted in psychology, medicine, AI and other fields, see, for example [44–46]. Such cases, which are often publicized as linked to a lack of reproducibility standards, are generating a crisis of trust within several scientific fields—which itself underlines the relationship we are discussing between information and trust [47].

## 5. External reliability

In addition to arguments that relate to facts as an outcome of a model or as a result of an experiment, it is important to stress that information is also the outcome of a *social* process.^9^ This simple and obvious message is too often forgotten or ignored.^10^ You could describe assessing the reliability of the research outcomes as a way of verifying whether these results have been obtained in a *rigorous* way, that is, whether a discipline-specific codification of certain rules has been strictly adhered to. But the external reliability of information—when it travels outside a specific research site, e.g. a laboratory, into a scientific journal or report—is verified in a different way.

To assess whether a paper can be published, the results are not empirically tested again but reviewed by *peers*. The peer-review process is clearly a social process, which can be faked. In addition, peer review does not establish the veracity of the published work, and some published work may contain errors still [52]. Who should be the peers also leads to problems of potential bias [53,54]. The Open Science reform movement involves many initiatives aimed at providing greater transparency of establishing exactly that external reliability, in addition to many other concerns addressed in Open Science [55]. That is, it includes initiatives to improve sharing of data, code and protocols but also of establishing research methodologies prior to performing the research, through for instance preregistration or registered reports.^11^ An interesting initiative in the context of misinformation is making academic work openly available as preprints prior to being accepted for publication by academic journals. In addition, preprints may be openly discussed in open peer-review procedures [56]. Initiatives such as pre-printing and open peer review might have dual effects. On the one hand, they help make the process more transparent, and thereby enhance the public trust people may have in science. On the other hand, making the discussion and potential disagreement among scientists more visible may reduce people’s trust in the scientific outcomes [57].

But facts also travel beyond the academic world, such as into the political domain. Their integrity can then be compromised because one part of a fact is what we know but the other part is what we do not know, the latter being expressed in terms of *uncertainty*.^12^^,^^13^ This is particularly salient in cases where knowledge is used to solve societal challenges. These cases—where knowledge is often uncertain, values are in dispute, stakes are high, and decisions are urgent—call for a high degree of *reflexivity* among both producers and users of knowledge [61,62]. Rather than waiting for science to deliver certainty, the focus shifts to intentional science and decision-making under uncertainty and value diversity [63]. Especially in the case of value conflicts in politics, knowledge producers may assume a different role than in less contentious settings [64]. This could include involving societal actors in science when their perspectives could provide a valid contribution, such as through extended peer-review, transdisciplinary research, knowledge co-production [65,66] or citizen science [67,68].

The role of science in society also requires explicitly dealing with uncertainty in scientific reporting and science communication. Uncertainty is challenging to communicate. It is often feared that uncertainty could decrease trust in the knowledge that is communicated. However, the impact of uncertainty on trust in facts seems to be small (negative or positive, if any), and uncertainty communication can increase trust in the communicator [69–72]. At the same time, a tendency among experts and politicians to refrain from acknowledging uncertainty and claims to ‘follow the science’ is also detrimental to public trust [73,74]. Knowledge may change as science progresses, and if the public and politicians were not made aware of this possibility, trust can be lost. Moreover, communicators of misinformation can easily highlight any obscured uncertainties and use this to increase the believability of their own information.

However, uncertainty information can be misused, insofar as it can be manipulated to sow unwarranted doubt—what Michaels & Monforton [75] called ‘manufactured uncertainty’, and other scholars term ‘manufactured doubt’ or ‘strategic use of uncertainty information’. For example, a specific uncertainty might be highlighted and used to cast doubt on the entire knowledge base, or uncertainties—which can rarely be fully removed—might be used as an argument to indefinitely postpone decisions (‘paralysis by analysis’).

Uncertainty, should be open for critical debate, until consensus is reached at some point. In other words, critical debate is part of the process of establishing facts. But not to sabotage it. One of the Merton’s well-known norms for science (CUDOs) is ‘organized scepticism’: scientific claims should be exposed to critical scrutiny before being accepted; both in methodology and institutional codes of conduct. However, this norm can also be abused to sabotage ever reaching consensus, and so that scientific results will never reach the status of fact [22].

Both obscuring and strategically magnifying uncertainty are examples of unreliable or irresponsible information practices. Good practices in uncertainty communication involve a more reflexive weighing of the information (facts and their uncertainties) and connecting this process to the needs of the potential users of this information. Furthermore, such communication should attempt to avoid potential misinterpretation and where necessary guide the user in the interpretation. For example, knowledge users can find it difficult to assess the relevance of uncertainty information; to distinguish which uncertainties are mostly academic details and which ones could have implications for interpreting the facts (e.g. robustness of the results) and for policymaking [76]. In many discussions around science in the context of societal challenges—often topics on which disinformation, misinformation and malinformation emerge—uncertainty is a fact of life, and society will need to find ways to make decisions under uncertainty. Despite the challenges, responsible communication of uncertainties—balanced and mindful of the needs of users of this information—is key to providing trustworthy information in this context.^14^

## 6. There are no bare facts

As mentioned earlier, statistical inference from data to facts is model-based, where a model can be anything ranging from mathematical equations, diagrams, graphs to physical objects. This inference serves to make sense of the data. As we start to gather evidence about a new phenomenon, such data do not make sense by themselves. They need to be organized and processed to see whether they show some patterns or some interrelations. This processing is usually done by translating the relevant data into a common medium (such as a model) where they can be examined for patterns, compared directly, and meaningful relations can be established between them.

Recent work has shown that narratives play an important role as a sense-making technology for science. This is especially the case when the data are not homogeneous or structured. According to Morgan [81], the quintessential feature of narrative is that it shows how things relate together, so that constructing a narrative account of a phenomenon in science involves figuring out how the supposed elements of a phenomenon can be related to each other to form a coherent account of that phenomenon. ‘Narrativizing serves to join things up, glue them together, express them in conjunction, triangulate, splice/integrate them together (and so forth)’. As such, narrative-making can be understood as a colligating process, that is, the binding together of a wider set of materials, not just of facts or evidence, but of hypotheses and ideas about a phenomenon.

The problem is deciding which narrative to privilege. In a symposium on the misuse of history, Forget [82] made clear that ‘we can correct error related to particular historical events, but we delude ourselves if we believe that a definitive history is possible’. It is important to have the facts correct, but these facts are not the most important part of history. Her contribution is to show that ‘history is about identity’. History confers identity because historical facts allow more stories to be told.

Facts can also be assessed in relation to their purpose or role in society. Societally relevant research is done for a variety of reasons (from general public interest to helping solve a specific problem) and is communicated to and used by specific audiences. It might play a role in public education, societal debate, policymaking or specific practical applications. One could question whether the information is fit for the purpose for which it is used. Literature on science–policy interfaces captures this in the notion of *knowledge quality*. High-quality knowledge is credible (scientifically adequate), salient (relevant to the issue at hand), legitimate (produced in a responsible way) and usable (practically applicable) [65,83,84]. The meaning of such criteria depends on the situation of course. For applied research, usable might mean that a city can use it to design its neighbourhoods or sewer systems; for basic research, it might mean that it can be used to improve existing models. The context and processes of knowledge production are as important for determining the external validity as the final information ‘product’. Low-quality information might be generated and communicated in isolation, without any regard for context, process or the needs of users and society at large; high-quality information is developed by keeping the context in mind from the start. Consider a scientific advisor who generates facts that support a client to obscure health effects of a consumer product: that would support the needs of the client (at least short term) but would be detrimental to society. For the purpose of assessing disinformation, high-quality information might be information that is developed and communicated in a way that is responsible and mindful of the context and use of this information.

## 7. Concluding remarks

We have discussed (dis)information in its relation to trust, not to truth. The trust in scientific information is related to the conditions under which information is generated and disseminated. In our overview of trust in science, we have shown how vulnerable the relationships between trust and information can be. We argue that we need to focus on restoring trust in science, for instance, by increasing transparency, broader inclusion and engagement with society, as argued for by Open Science. At the same time, we should clarify what sources are untrustworthy, not because their results are wrong or incorrect, but because their procedures cannot be trusted.

In this article, we focused on scientific information, that is facts in terms of outcomes of scientific research that meet certain procedural standards of scientificity. However, such procedures are not watertight, and having certain standards only induce trust and is therefore not exhaustive or complete. Facts established through such procedures can later turn out to be mistakes. If we learn from these mistakes, this would be a hallmark of science and a cornerstone of scientific procedures.^15^

The consequence of this is that debunking, i.e. correcting verifiable falsity and fact-checking, although valuable, cannot address the fundamental problem with scientific misinformation. Instead, we should aim to clarify whether or not certain standards for procedures are met by certain sources. By clarifying these shortcomings, citizens are better equipped to spot and mistrust disinformation.

## Data Availability

This article has no additional data.

## Footnotes

^1^
To discuss trust, we use several related terms, such as reliability, trustworthiness and validity. While we have been careful in their use and clarified them where necessary, some ambiguity remains inevitable. The reason for this is that we aimed to stay close to the terminology used in the different contexts we discuss in this article resulting in a slight variation in meanings between these contexts.

^2^
The more technical definition used in philosophy of information is also not very helpful in combating disinformation because it adopts the veracity thesis that information is true by definition. The problem with this definition is that whenever something is claimed to be information, one must indeed still verify its veracity.

^3^
A good example of the opposite is [8].

^4^
See [12] for an overview of current individual-level interventions.

^5^
While we continue to use the EU definition [21], we find its implication that disinformation is harmful by definition to be noteworthy. One might think that certain information could be considered disinformation even if it would have no harmful consequences. It would only reduce the need to intervene in harmless disinformation.

^6^
This is not to say that models do not play a role in experiments. See [35] for a classic argument about this.

^7^
This idea of integrity of facts is borrowed from [37].

^8^
A critical discussion of reproducibility as an overarching epistemic value, however relevant today, takes us too far beyond these scope of this paper. See [38,39] for a detailed discussion.

^9^
The social construction of scientific facts is most famously discussed by [48].

^10^
An influential reminder of this ‘Myth of the Given’ was [49]. One of the first reminders in economics is [50]. A most recent reminder in economics is [51].

^11^
It should be noted that preregistration also relies upon peer review, albeit of course at a more constructive phase of the research. We would like to thank Heather Douglas for highlighting this.

^12^
In measurement science, uncertainty ‘reflects the lack of knowledge of the value of the measurand’ [58, p. 25]. Although the sources of uncertainty are similar to the sources of error, in measurement science the term ‘uncertainty’ is preferred rather than ‘error’. The reason is that error is defined in its relation to truth, but ‘true values are by nature indeterminate’ [59, p. 32], it would require complete knowledge of the measurand and so an ‘infinite amount of information’ [59, p. 49].

^13^
In research on science-policy interfaces, Walker *et al.* [60] define uncertainty from the point of view of scientists providing (model-based) information to support policymaking as ‘any deviation from the unachievable ideal of completely deterministic knowledge of the relevant system’, distinguishing three dimensions: location, level and nature. Note that they argue that different actors (modellers, policymakers, citizens, etc.) may have different views on uncertainty, and that some uncertainties can be reduced through more research but others cannot.

^14^
Various science-policy boundary organizations have also developed good practice guidances on uncertainty communication and responsible handling of uncertainty in general (e.g. [77–80]).

^15^
This is of course the core of Popper’s critical rationalism. See [85] for a more recent application.
